# Catabolic pathway acquisition by rhizosphere bacteria readily enables growth with a root exudate component but does not affect root colonization

**DOI:** 10.1128/mbio.03016-24

**Published:** 2024-12-11

**Authors:** Stephan Christel, Alyssa A. Carrell, Leah H. Hochanadel, Manuel I. Villalobos Solis, Paul E. Abraham, Sara S. Jawdy, Julie E. Chaves, Nancy L. Engle, Timkhite-Kulu Berhane, Tao Yao, Jin-Gui Chen, Wellington Muchero, Timothy J. Tschaplinski, Melissa A. Cregger, Joshua K. Michener

**Affiliations:** 1 Biosciences Division, Oak Ridge National Laboratory, Oak Ridge, Tennessee, USA; University of California, Irvine, Irvine, California, USA

**Keywords:** horizontal gene transfer, plant-microbe interactions, *Populus*, salicylates

## Abstract

Horizontal gene transfer (HGT) is a fundamental evolutionary process that plays a key role in bacterial evolution. The likelihood of a successful transfer event is expected to depend on the precise balance of costs and benefits resulting from pathway acquisition. Most experimental analyses of HGT have focused on phenotypes that have large fitness benefits under appropriate selective conditions, such as antibiotic resistance. However, many examples of HGT involve phenotypes that are predicted to provide smaller benefits, such as the ability to catabolize additional carbon sources. We have experimentally simulated the consequences of one such HGT event in the laboratory, studying the effects of transferring a pathway for catabolism of the plant-derived aromatic compound salicyl alcohol between rhizosphere isolates from the *Pseudomonas* genus. We find that pathway acquisition enables rapid catabolism of salicyl alcohol with only minor disruptions to the existing metabolic and regulatory networks of the new host. However, this new catabolic potential does not confer a measurable fitness advantage during competitive growth in the rhizosphere. We conclude that the phenotype of salicyl alcohol catabolism is readily transferable but is selectively neutral under environmentally relevant conditions. We propose that this condition is common and that HGT of many pathways will be self-limiting because the selective benefits are small.

**IMPORTANCE:**

Horizontal gene transfer (HGT) is a key process in microbial evolution, but the factors limiting HGT are poorly understood. Aside from the rather unique scenario of antibiotic resistance, the evolutionary benefits of pathway acquisition are still unclear. To experimentally test the effects of pathway acquisition, we transferred a pathway for catabolism of a plant-derived aromatic compound between soil bacteria isolated from the roots of poplar trees and determined the resulting phenotypic and fitness effects. We found that pathway acquisition allowed bacteria to grow using the plant-derived compound in the laboratory, but that this new phenotype did not provide an advantage when the bacteria were reinoculated onto plant roots. These results suggest that the benefits of pathway acquisition may be small when measured under ecologically-relevant conditions. From an engineering perspective, efforts to alter microbial community composition *in situ* by manipulating catabolic pathways or nutrient availability will be challenging when gaining access to a new niche does not provide a benefit.

## INTRODUCTION

Due to competing processes of gene gain by horizontal gene transfer (HGT) and gene loss, bacterial gene content can vary widely even between strains of the same species (1, 2). These “accessory” genes, which are only present in a subset of strains, often alter the potential niche of the host (3), for example by encoding pathways to assimilate additional nutrients or tolerate new stresses (4, 5). However, the fitness effects of accessory pathway acquisition are unclear, with arguments both for models that are largely adaptive or largely neutral (6, 7).

Any such discussion of fitness effects must include both the benefits of pathway acquisition as well as the associated costs (8, 9). The fitness effect of an accessory gene depends on a broad range of interactions, both with its host and the environment (10). For example, the acquisition of a pathway that provides access to a new niche will only be beneficial if the costs of pathway acquisition and integration are low while the benefits of niche expansion are high. These costs and benefits will depend on details of the genome content of the host strain and the biotic and abiotic conditions of the environment that the host inhabits.

The costs and benefits from HGT are often analyzed through knockout studies, removing putative horizontally-transferred genes and measuring changes in phenotype or growth (11). However, these experiments are confounded by historical evolution following gene transfer, which can mitigate the costs of newly-acquired genes or introduce new dependencies (12, 13). The effects of pathway acquisition, for example by HGT, can be more directly assessed by targeted pathway transfer in the laboratory followed by analysis of changes in phenotype and fitness (14).

If the benefits of HGT outweigh the costs, then targeted pathway transfer provides a potential opportunity to deliberately manipulate bacterial colonization, with applications in health, agriculture, and environmental remediation. For example, generating a new niche by feeding a marine polysaccharide to mice allowed specific colonization by a bacterium that had been engineered to contain the associated catabolic pathway (15). In this case, the costs of pathway acquisition were low and the benefits were high. In general, this situation is likely to be rare, as many such ecological niches will already be filled by native microbes (16).

Even when an open niche is available, the utility of gaining access to a new niche may be small if the host already has access to other niches. For example, a recalcitrant environmental pollutant is an open niche that could be exploited as a carbon and/or energy source. However, when introducing allopatric microbes for bioremediation, metabolic specialists are more successful than generalists, likely because they have fewer alternative niches available in their new environment (17, 18).

Similar to the gut and soil, the rhizosphere is a complex environment with abundant metabolic niches and opportunities for HGT (19). Plant root exudates provide diverse carbon sources that support high microbial populations (20). Metabolic cross-feeding and spatial structure further broaden the range of available niches (21). These factors are particularly significant in perennial plants that can maintain a dynamic microbiome across multiple seasons (22).

Poplar (*Populus* sp.) trees provide a tractable model system to study microbial dynamics in the perennial rhizosphere (23). Poplar trees exude large quantities of phenolic compounds derived from salicyl alcohol (SA), including salicin, populin, and tremuloidin (24). These compounds are thought to act primarily as inhibitors of herbivory (25) but also serve as potential carbon sources for soil microbes (26). An intermediate in the SA catabolic pathway, salicylic acid is a major component of plant exudates and is thought to influence microbiome community composition (27). Therefore, SA is a representative microbial metabolic niche in the rhizosphere, and HGT of pathways for SA catabolism is likely to occur frequently. The persistence of these pathways after transfer will depend on the costs and benefits of pathway acquisition. Pseudomonads are abundant members of the rhizosphere microbiome and are known for their aromatic catabolic potential, making them likely donors and recipients of SA catabolic pathways (28, 29).

**Fig 1:**
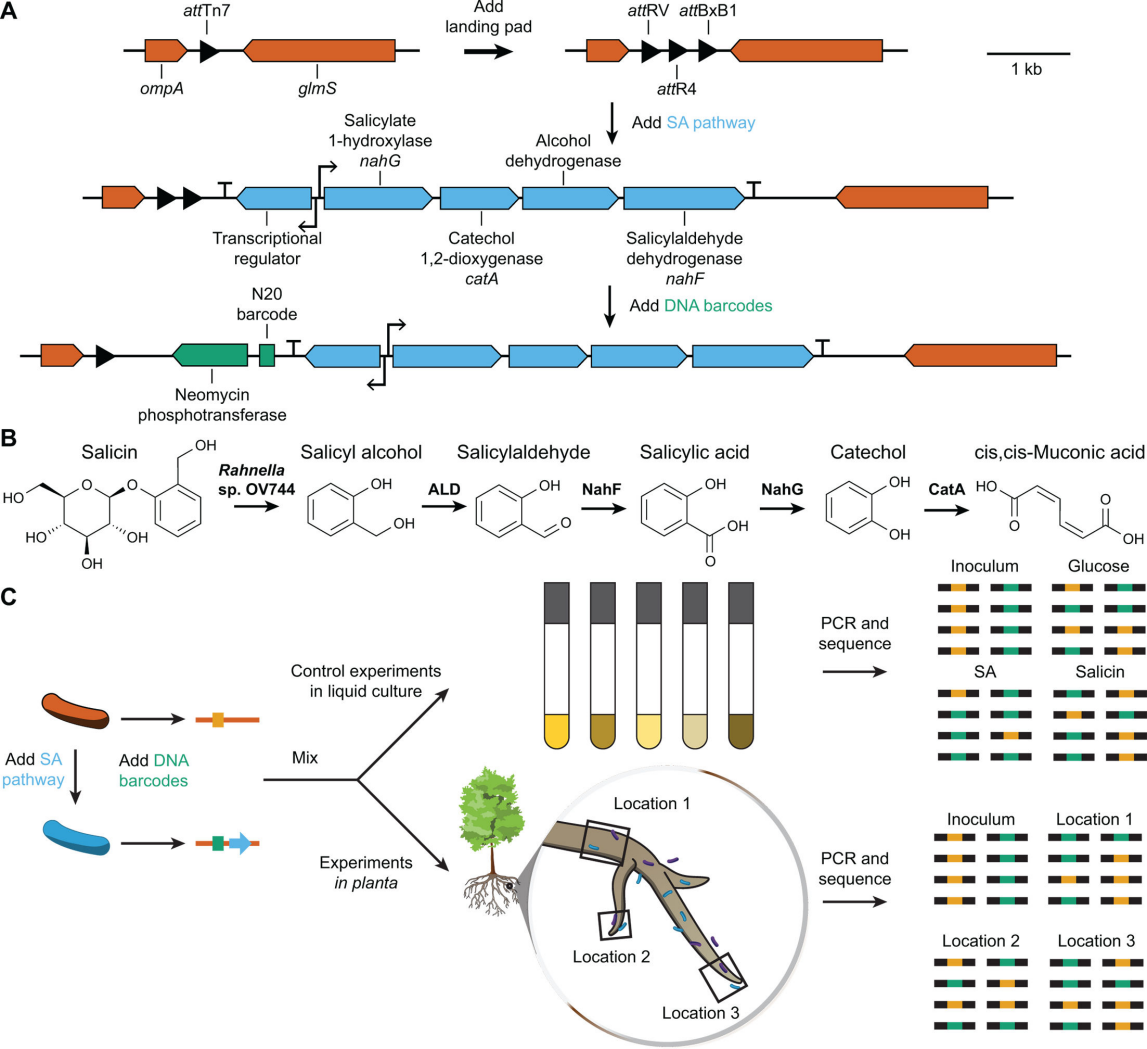
Experimental overview. (**A**) To mimic HGT, the SA catabolic pathway from *Pseudomonas* sp. GM16 was transferred to the Tn7 *att* site in other *Pseudomonas* strains. DNA barcodes were then introduced to allow strain tracking *in situ*. (**B**) The proposed pathway for salicin degradation is initiated by a glycosyltransferase in a complementary strain, such as *Rahnella* sp. OV744, followed by successive oxidation to *cis*,*cis*-muconic acid. The tested *Pseudomonas* strains contain native pathways for the assimilation of muconic acid. (**C**) Barcode amplicon sequencing was used to measure the fitness effects of pathway acquisition, both in liquid culture and in the rhizosphere.

In this work, we have assessed the utility of acquiring a pathway for catabolism of SA (Fig. 1A). We show that salicyl alcohol catabolism is common among strains isolated from the *Populus* rhizosphere and this phenotype can readily be transferred through genetic engineering into strains that do not natively possess it. The fitness costs and physiological disruption due to pathway acquisition are small. However, root colonization assays show that the fitness benefits are also minimal, even under conditions designed to maximize these effects. We conclude that the acquisition of this catabolic pathway is functionally beneficial, in that it provides new capabilities at minimal cost, but selectively neutral.

## RESULTS AND DISCUSSION

### Pseudomonad growth with salicyl alcohol

**Fig 2:**
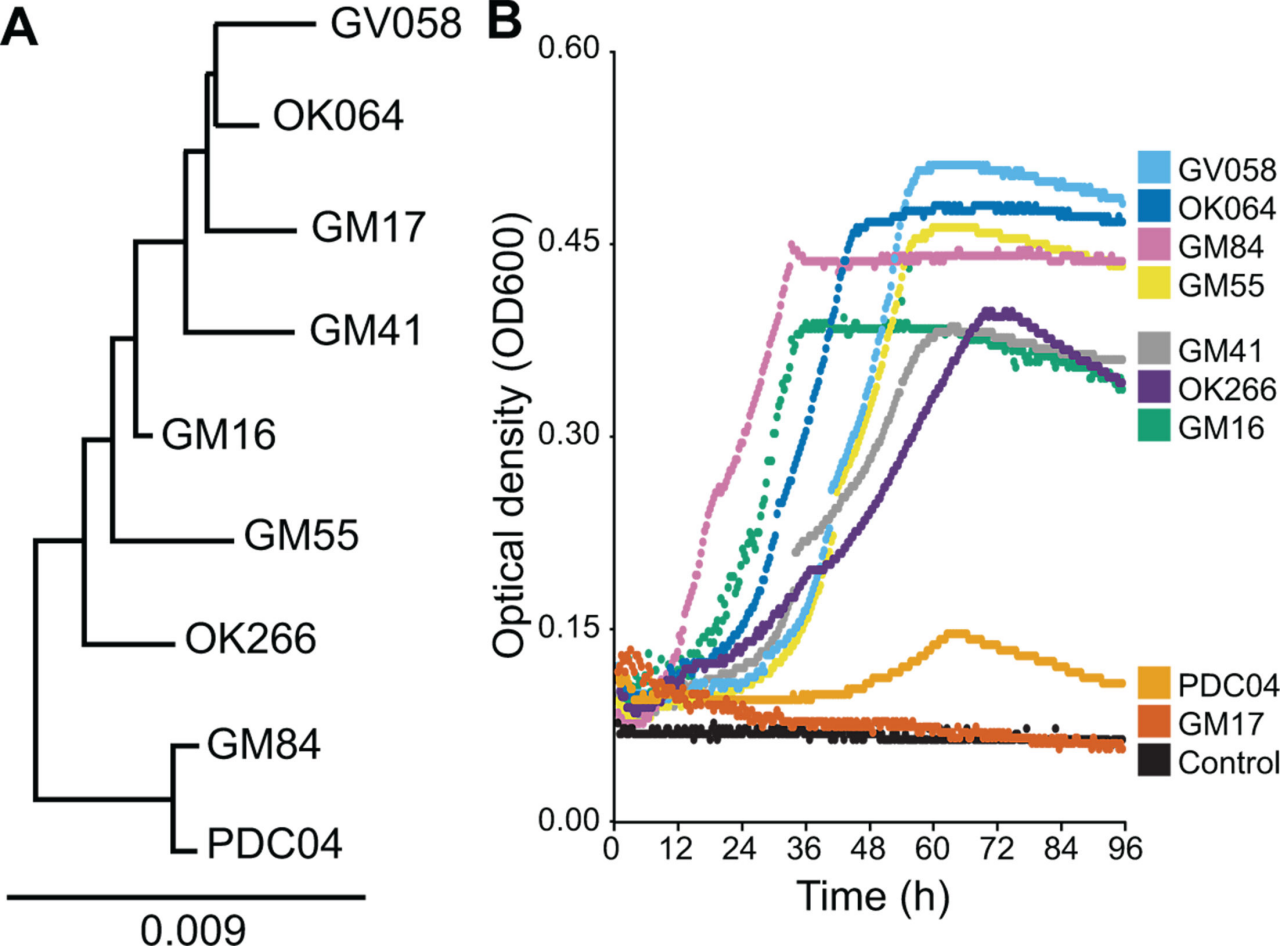
Salicyl alcohol catabolism is common among soil *Pseudomonas* isolates. (**A**) 16S phylogenetic tree of *Pseudomonas* strains isolated from *Populus* and tested for SA catabolism. *Cellovibrio japonicus* was used as an outgroup (not shown). (**B**) Growth curves of *Pseudomonas* strains in M9 minimal medium with salicyl alcohol as the sole source of carbon and energy. One representative curve is shown for each strain, chosen from three biological replicates.

To evaluate the frequency of SA catabolism in rhizosphere pseudomonads, we tested for growth with SA among nine diverse *Pseudomonas* strains previously isolated from *Populus* roots (Fig. 2A) (30). Eight of the isolates grew in M9 minimal medium with SA as the sole source of carbon and energy, including the previously characterized *Pseudomonas* sp. GM16 (hereafter “GM16”) (26). One strain, *Pseudomonas* sp. GM17 (hereafter “GM17”), did not grow under these conditions (Fig. 2B).

Given the high frequency of SA catabolism in these isolates, we sought to understand the factors limiting the dissemination or retention of this pathway in GM17. We initially hypothesized that deleterious interactions between a newly introduced SA pathway and the native metabolic pathways of the potential hosts would prevent successful transfer into GM17 (31, 32). To test this hypothesis, we engineered the SA catabolic pathway into several *Pseudomonas* isolates and measured changes in catabolic activity. We chose GM17 as a representative non-catabolizing soil isolate, *Pseudomonas putida* KT2440 as a non-catabolizing laboratory reference strain, and *Pseudomonas* sp. PDC04 as a representative poorly catabolizing isolate. We transferred the SA catabolic pathway from *Pseudomonas* sp. GM16 (26) into these strains, as homologous pathways are found in the chromosomes of distantly related *Pseudomonas* sp. OK266 and *Pseudomonas* sp. PDC04 (Fig. S1) but not in more closely related strains, likely indicating prior HGT of this pathway. While PDC04 contains a homologous SA catabolism pathway, it grows poorly with SA and we hypothesized that the acquisition of an additional highly active pathway might improve growth.

**Fig 3:**
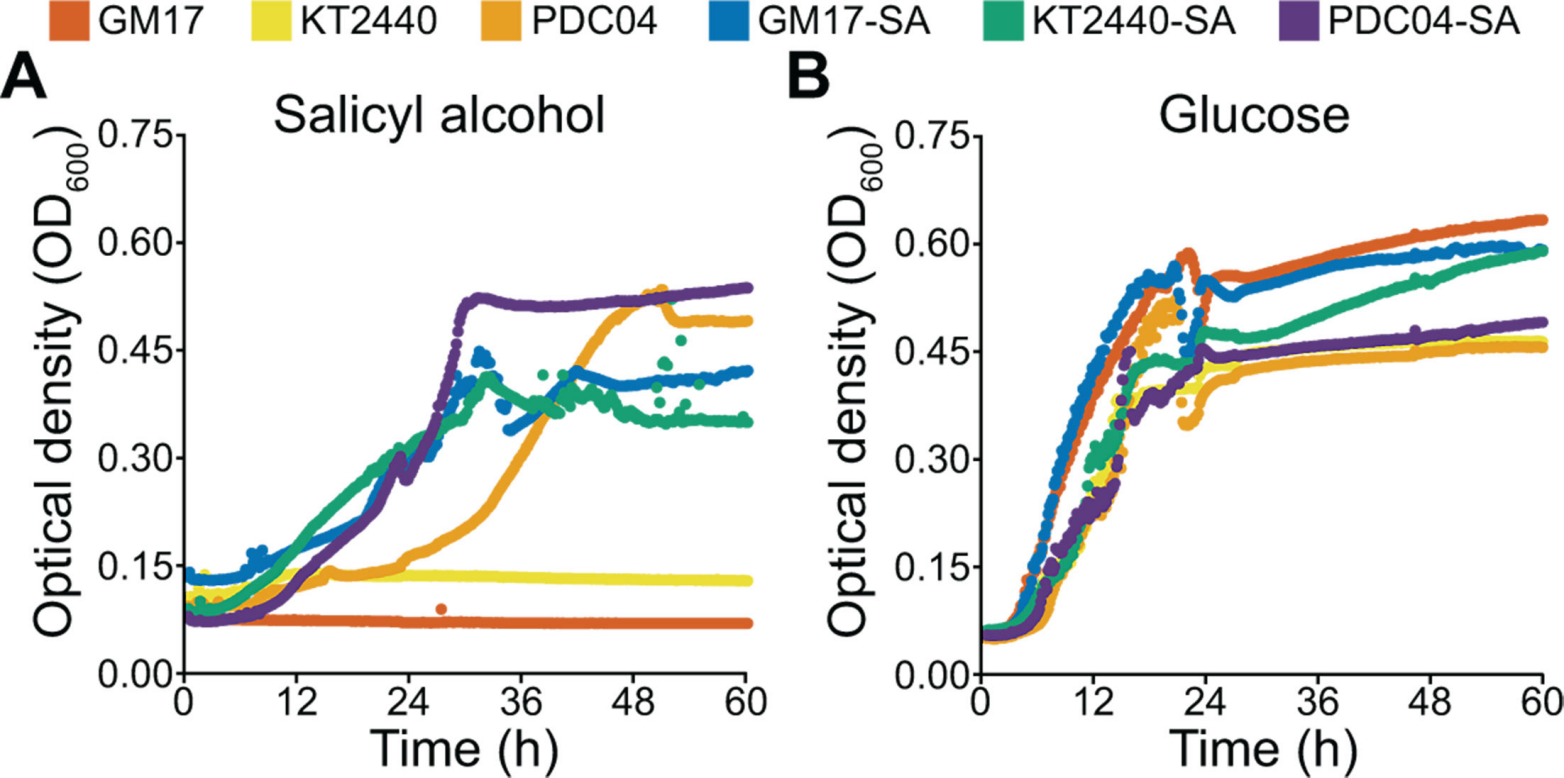
Growth of wild-type and engineered *Pseudomonas* strains. The “SA” prefix indicates that the strain contains a genomically-integrated SA catabolic pathway. Individual strains were grown in M9 minimal medium containing (**A**) 1 g/L salicyl alcohol or (**B**) 1 g/L glucose as the sole carbon and energy source. One representative curve is shown for each strain, chosen from three biological replicates.

We first integrated *attP* sites for heterologous serine integrases into the T7 phage integrase *att* site in each recipient strain (33–35). We then used the heterologous BxB1 *attP* site to stably introduce the SA catabolic pathway from GM16, including its putative SalR regulator, into the genomes of the recipients. When we measured growth with SA, we found that all three engineered strains could now grow with SA (Fig. 3A). Strain PDC04, which could naturally metabolize SA, grew more rapidly with SA after the introduction of the heterologous catabolic pathway. We did not observe noticeable changes in growth with glucose between the wild-type and engineered strains (Fig. 3B).

### Proteomics analysis of pathway integration

While the engineered strains grew readily with SA, we hypothesized that pathway acquisition might impose subtle stresses on the new host that would limit pathway retention under more stringent selective conditions (36, 37). To test this hypothesis, we performed a global proteomic analysis of one wild-type strain, GM16, and three engineered strains, GM17-SA, PDC04-SA, and KT2440-SA. Each strain was grown with SA as the sole carbon source and compared to the corresponding strain grown with glucose.

On average, 27.7% ± 2.6% of measured proteins were detected at significantly (q < 0.05 and log_2_ fold-change > 2) higher or lower abundance during growth with SA (Table 1). This broad shift in expression could indicate a relatively large physiological perturbation. However, based on gene annotations, the vast majority of differentially expressed proteins were likely directly involved in the degradation of SA and its cascading products.

**TABLE 1:** Differentially abundant proteins identified in analysis of four wild-type/SA strain pairs grown in triplicate in the presence of 0.1% glucose versus 0.1% salicyl alcohol^*a*^

Strain	GM16	KT2440	GM17	PDC04
Total detected proteins	2,069	1,891	2,070	1,903
Upregulated glucose	217	238	172	245
Upregulated SA	277	351	389	303
Total differentially abundant proteins	494	589	561	548
Percentage of total	23.9	31.1	27.1	28.8

^*a*^ *P* < 0.05 and log_2_ fold-change > 2.

**Fig 4:**
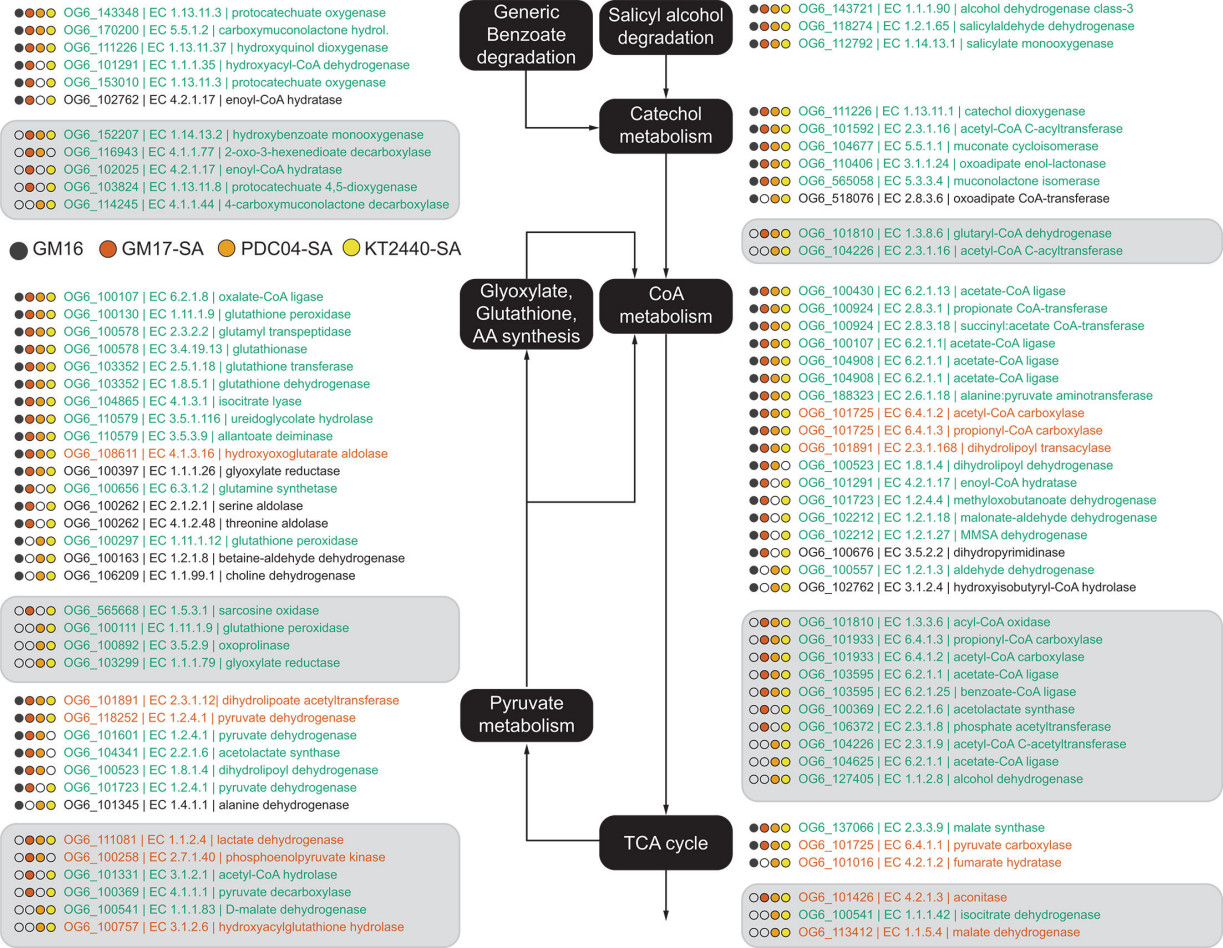
Proteins with significantly increased abundance in wild-type GM16 and engineered GM17, PDC04, and KT2440 strains when grown with SA compared to glucose. Filled circles indicate that the orthologous group was identified to be significantly differentially expressed in the corresponding strain during growth with SA versus glucose. Orthologous groups were grouped according to their predicted function and displayed along the pathway of SA oxidation (black boxes). Green text indicates increased expression in the presence of SA, red text decreased expression, and black text contradicting expression in the strains. Light gray background indicates orthologous groups uniquely identified in the mutants.

We used OrthoMCL to group and compare protein functions rather than often misleading sequence identity. This analysis revealed that all but a few significantly differentially abundant proteins could be placed along the flow of carbon from SA to acetyl/succinyl-CoA metabolism and TCA cycle (Fig. 4) and that this behavior was indeed shared between all three SA strains plus the original host of the SA degradation pathway, GM16. Very few ortholog groups shared differential expression patterns in all engineered strains but not GM16. Among them was a hydroxybenzoate monooxygenase (Orthogroup OG6_152207), a glutaryl-CoA dehydrogenase (OG6_101810), as well as several dehydrogenases, carboxylases, and ligases involved in CoA and pyruvate metabolism (Fig. 4). The lack of such a distinct “mutant signature,” i.e., the absence of a large set of differentially abundant proteins common exclusively to engineered strains, indicated that the introduced pathway did not disrupt genetic regulation in its new host strains. Furthermore, detailed abundance analysis of the four proteins newly introduced into the engineered strains revealed that they were only minimally expressed during growth on glucose (data not shown), confirming that the regulatory systems from GM16 also remained functional. Based on the proteomics results, we concluded that the pathway is active, properly regulated, and does not cause significant stresses to the new host bacteria.

### Impact of pathway acquisition on root colonization

Since the SA pathway was functional and minimally disruptive, we next tested its effect under more environmentally-realistic conditions, during colonization of *Populus trichocarpa* roots. To track changes in relative bacterial abundance, we introduced random 20 nt DNA barcodes flanked by conserved primer binding sites into the R4 *attB* sites in wild-type and engineered GM17 (Fig. 1A). We downsampled each library to approximately 10,000 barcodes per strain and sequenced each library to identify the barcodes that uniquely identified each strain. Changes in the relative abundance of the two strains can then be tracked by targeted amplicon sequencing of the barcode region.

To test the accuracy of the assay, we mixed the barcoded populations of wild-type and engineered GM17 and grew the mixed culture in liquid culture with glucose or salicyl alcohol as the sole carbon source. We sequenced amplicons from the inoculum and saturated cultures and determined changes in the relative abundance of the wild-type and mutant strains (Fig. S2). We observed no change in relative abundance of the SA mutant after growth with glucose, but an enrichment for the SA mutant after growth with SA. These results are consistent with prior growth experiments using pure cultures, showing that the SA pathway is active and provides a growth advantage when SA is the sole carbon and energy source but imposes a minimal fitness cost during growth with glucose. To determine the sensitivity of this assay, we also used the mixed culture to inoculate roots of tissue-cultured *Populus trichocarpa* grown in sterile clay. We grew the resulting plants for 21–28 days before harvesting the trees. We then dissected the roots into segments ranging in mass from approximately 10 mg to less than 0.1 mg (Fig. S3) and performed amplicon sequencing on the barcodes. We reliably amplified barcodes from root segments with masses less than 1 mg (Fig. S4).

**Fig 5:**
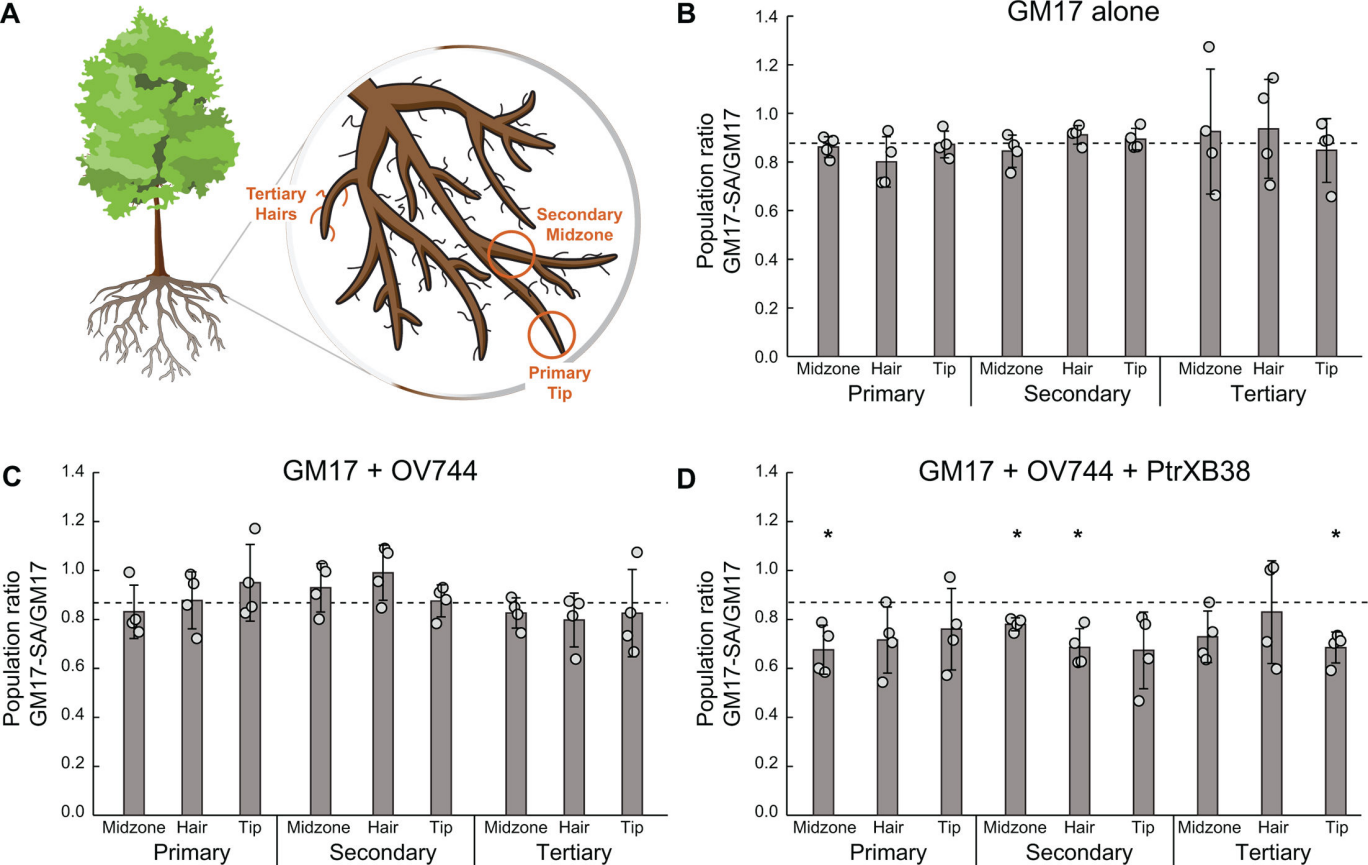
SA catabolism provides no advantage during plant colonization. (**A**) Mixtures of bacteria were inoculated onto axenic *P. tremula* x *P. alba* “INRA 717-1B4” cuttings and grown for 28 days. Population ratios were sampled at a range of sites, including primary/secondary/tertiary roots at the tip/midzone/root hairs. Representative sites are shown in red. (**B**) A mixture of wild-type and engineered GM17 was inoculated onto axenic *P. tremula* x *P. alba* “INRA 717-1B4”. The population ratios before and after cultivation were determined by barcode amplicon sequencing. The dashed line shows the population ratio of the inoculum. Error bars show one standard deviation, calculated from the four biological replicates shown. (**C**) Same as B, but with the addition of the salicin-degrading bacterium *Rahnella* sp. OV744. (**D**) Same as C, but using a salicin-overproducing *PtrXB38-OE* line of *P. tremula* x *P. alba* “INRA 717-1B4.” *, Final population ratio differs significantly from the inoculum (*P* < 0.05). All other comparisons are non-significant.

Next, we inoculated tissue-cultured *Populus tremula* x *Populus alba* “INRA 717-1B4” with the GM17 mixed culture and measured changes in relative abundance. We hypothesized that, if the SA pathway provided a fitness advantage to its host during colonization, then the abundance of barcodes from the engineered strain would increase relative to the wild-type strain (Fig. 1C). We did not add any exogenous source of carbon to these experiments, so plant root exudates are expected to be the only carbon available to the bacteria. Since plant metabolite profiles are expected to vary spatially based on root architecture, we tested replicate samples from primary, secondary, and tertiary root segments, root hairs, and root tips (Fig. 5A). However, we observed no significant differences in abundance in any location (*P* < 0.05) (Fig. 5B). The statistical power (β > 0.2, *n* = 4, d = 2.38) was sufficient to detect changes in the population ratio outside the range 0.74–1.02, corresponding to an estimated competitive fitness for the mutant relative to wild type of 0.91–1.09. We concluded that the presence of the SA pathway was providing a minimal net fitness benefit, either because the gross benefits were small or because there was a corresponding cost to SA pathway maintenance and expression.

### Effects of epistatic interactions that benefit SA catabolizers

While *Populus* secretes small amounts of SA, it primarily secretes SA conjugates, including salicin (Fig. S5). Neither wild-type nor engineered GM17 can degrade salicin, which might limit the effect of SA catabolism in the absence of an accessory deglycosylation pathway. Since the co-cultures of GM16 and *Rahnella* sp. OV744 (hereafter “OV744”) has been shown to fully degrade salicin, we tested whether the presence of OV744 would alter the fitness effect of SA catabolism in GM17. We first repeated the *in vitro* assays, inoculating wild-type, and engineered GM17 into an M9 minimal medium with salicin as the sole carbon source in the presence and absence of OV744. Using amplicon sequencing to specifically track changes in the abundance of wild-type and engineered GM17 from the mixed culture, we observed that the SA pathway provided a fitness benefit during growth with salicin only when OV744 was also present in the culture (Fig. S2). We also observed a small fitness cost for the SA pathway during growth with glucose in the presence of OV744, which was not observed in the absence of OV744. These results are consistent with the model that OV744 converts salicin into SA, which is then available for catabolism by the GM17 SA mutant (Fig. 1B).

We repeated the *P. trichocarpa* inoculations using the GM17 mixed library in the presence of OV744 and sampled a range of root positions after 28 days of growth. We again observed no significant changes in the relative abundance of the wild type versus engineered GM17 strains (Fig. 5C). The estimated fitness of the mutant relative to the wild type was within the range 0.82–1.16 (β > 0.2). By comparison to the control experiments, we conclude that SA production from salicin by OV744 was too low to provide a measurable fitness benefit to the GM17 SA mutant.

To further amplify the potential benefits of SA catabolism, we repeated these experiments using a *P. tremula* x *P. alba* INRA 717-1B4 mutant that overexpresses the *PtrXB38* gene (38). This mutant has been shown to produce significantly higher concentrations of salicin in the roots (Fig. S5). However, as in our previous experiments, we saw no enrichment for the SA-degrading GM17 strain during growth with the *PtrXB38-OE* plants either in the presence or absence of OV744 (Fig. 5D; Fig. S6). The only statistically significant changes in population ratio were slight decreases in the abundance of GM17-SA in four out of nine compartments, which were not observed in the absence of OV744 (Fig. S6). The salicin concentration is higher in the *PtrXB38-OE* plants, which would presumably provide more SA after hydrolysis by OV744. However, the concentration may still be too low compared to the other available carbon sources to provide a significant fitness difference. Alternatively, the public benefits of SA detoxification provided by GM17-SA to wild-type GM17 may outweigh the private benefits of SA catabolism.

### Conclusions

In combination, our results demonstrate that the acquisition of a pathway for SA catabolism, either in the laboratory through metabolic engineering or presumably in nature through HGT, can readily provide rhizosphere isolates with new metabolic capabilities. The pathway for SA catabolism imposes minimal disruption on the native pathways of the new host bacterium. However, SA catabolism did not provide a fitness benefit during root colonization, even under conditions that are designed to favor SA catabolizers.

We hypothesize that, under the conditions tested, GM17 can access sufficient metabolic niches so that the availability of a new niche does not alter its fitness. In a more realistic microbial community, it might face additional competition in these other niches and gain a larger proportional advantage from SA catabolism. However, as we have shown, a more realistic community would also contain many other SA-catabolizing microbes, so the benefits of the new niche would also be smaller.

In general, our results are consistent with a model where catabolic pathways spread through HGT until the benefits of pathway acquisition decline to the point that they are balanced by the small costs of pathway maintenance. While catabolic potential can drive significant changes in colonization, we suggest that those examples will be limited to rare metabolites that are not efficiently exploited by the native microbiota and specialist microbes that have few alternative niches available. These conclusions are consistent with prior evidence from bioaugmentation for bioremediation and offer cautionary guidance for efforts to control the colonization of introduced microbes into native communities through engineering carbon utilization.

## MATERIALS AND METHODS

### Strains

*Pseudomonas* strains used in this study were *Populus*-derived isolates from the ORNL Plant-Microbe Interfaces strain collection (30). *P. putida* KT2440 was acquired from the American Type Culture Collection. Genome sequences (complete or partial) are available for all used wild-type strains and can be retrieved from online repositories (Table 2).

**TABLE 2:** Strains used in this study

Strain	Genotype	Reference
*Pseudomonas* sp. GM16	Wild type	(30)
*Pseudomonas* sp. GM17	Wild type	(30)
*Pseudomonas* sp. PDC04	Wild type	(30)
*Pseudomonas putida* KT2440	Wild type	ATCC
*Rahnella* sp. OV744	Wild type	(30)
*Pseudomonas* sp. GM41	Wild type	(30)
*Pseudomonas* sp. GM55	Wild type	(30)
*Pseudomonas* sp. GM84	Wild type	(30)
*Pseudomonas* sp. GV054	Wild type	(30)
*Pseudomonas* sp. GV058	Wild type	(30)
*Pseudomonas* sp. OK064	Wild type	(30)
*Pseudomonas* sp. OK266	Wild type	(30)
JMP43	GM16 *att*Tn7::*att*BxB1/*att*RV/*att*R4	This work
JMP44	GM17 *att*Tn7::*att*BxB1/*att*RV/*att*R4	This work
JMP52	PDC04 *att*Tn7::*att*BxB1/*att*RV/*att*R4	This work
JMN42	KT2440 *att*Tn7::*att*BxB1/*att*RV/*att*R4	This work
JMP88	GM17 *att*Tn7::(*att*BxB1::SA-Reg)/*att*RV/*att*R4	This work
JMP90	PDC04 *att*Tn7::(*att*BxB1::SA-Reg)/*att*RV/*att*R4	This work
JMP91	KT2440 *att*Tn7::(*att*BxB1::SA-Reg)/*att*RV/*att*R4	This work
JMP96	GM17 *att*Tn7::*att*BxB1/*att*RV/(*att*R4::Barcodes)	This work
JMP97	GM17 *att*Tn7::(*att*BxB1::SA-Reg)/*att*RV/(*att*R4::Barcodes)	This work

### Construction of a broad host range tri-*attP* landing pad

A landing pad containing three phage integrase *attP* recognition sequences was designed for chromosomal integration using a broad host range mini-Tn7 vector method (33). First, the tri-*attP* landing pad sequence was cloned into a mini-Tn7 vector, followed by co-transformation into each *Pseudomonas* strain with a plasmid encoding Tn7 transposition pathway expression (Table 3). Finally, the antibiotic selection marker for the mini-Tn7 vector was removed by Flp-mediated excision. The mini-Tn7 vector (pUC18T-mini-Tn7T-GM, Addgene plasmid # 63121), Tn7 transposase expression plasmid (pTNS, Addgene plasmid # 64967), and the FLP recombinase expression plasmid (pFLP3, Addgene plasmid # 64946) were gifts from Herbert Schweizer (39).

**TABLE 3:** Plasmids used in this study

Plasmid	Genotype	Reference
pUC18T-mini-Tn7T-GM		(39) Addgene 63121
pTNS2		(39) Addgene 64968
pFLP3		(39) Addgene 64946
pJM442	pUC18T-mini-Tn7T-GM + *attB*R4/*attB*BxBI/*attB*RV	This work
pGW44	BxB1*attP colE1 kanR*	(35)
pJM455	pGW44 + SA catabolism pathway	This work
pJH207	R4*attP colE1 kanR*	(35)
pJM488	pJH207 + 20 nt barcodes	This work

The R4, Bxb1, and RV phage integrase *attP* recognition sequences were designed into a single landing pad sequence and synthesized *de novo* (Twist Biosciences). The landing pad sequence was PCR amplified from the vector, and cloned into the mini-Tn7 vector following manufacturer instructions (NEBuilder HiFi Assembly Master Mix) to generate a mini-Tn7 vector + Landing Pad plasmid pJM442.

### Construction of *Pseudomonas* recipient strains

Each wild-type *Pseudomonas* strain (Table 2) was transformed with pJM442 plasmid using quad-parental conjugation as previously described (33). *Escherichia coli* WM6062, a diaminopimelate (DAP) auxotroph strain carried the mini-Tn7 vector + Landing Pad plasmid (pJM442), *E. coli* Pir1 carried the pTNS2 Tn7 transposase expression plasmid, and *E. coli* DH5α carried a plasmid containing the conjugation machinery (pRK2073_Kan^R^). All four strains were grown overnight in LB medium supplemented with the appropriate antibiotics or nutrients (DAP), at 30°C (*Pseudomonas*) or 37°C (*E. coli*). Saturated cultures were combined into a single Eppendorf tube at equal 100 mL volumes. The mixed culture was then centrifuged at room temperature at 7,000 × *g*, washed twice in 1 mL of sterile 10 mM MgSO_4_, and resuspended into 30 uL 10 mM MgSO_4_. The final resuspension was dropped onto a pre-dried LB agar plate supplemented with DAP (60 mg/mL), and incubated overnight at 30°C. The next day, the cell biomass was scraped from the agar plate, resuspended into 5 mL sterile 10 mM MgSO_4,_ and serially diluted. Then, 100 µL of the 10^−3^ and 10^−5^ dilutions were spread onto LB agar plates supplemented with 100 µg/mL gentamicin without DAP and incubated at 30^o^C overnight or until clear colonies appeared. Proper integration of the landing pad into each strain was verified by whole-genome resequencing.

To complete the recipient strain construction, the gentamycin resistance cassette was removed from each strain using Flp-mediated excision. Chemically competent versions of each strain were generated (40). Single colonies of each strain were used to inoculate LB medium supplemented with 100 µg/mL gentamycin and grown to saturation overnight. Saturated cultures (1 mL) were transferred to pre-chilled Eppendorf tubes and centrifuged at room temperature, for 1 min at 13,000 *× g*. The supernatant was decanted, and the pellet was resuspended and washed twice in 1 mL cold 0.1 mM MgCl_2_ at room temperature. After the second wash, the pellet was resuspended with 1 mL cold TG salt (75 mM CaCl_2_, 6 mM MgCl_2_, and 15% glycerol), incubated for 10 min on ice, centrifuged as above, and resuspended with 200 µL TG salt. The cells were then flash frozen in liquid nitrogen, and kept at −80°C until used for transformation.

Aliquots of 100 µL of each chemically competent *Pseudomonas* strain were mixed with 100 ng of the FLP recombinase expression plasmid (pFLP3), incubated together on ice for 15 min, followed by a 2-min heat shock at 37°C. Cells were immediately resuspended in 900 µL SOC and allowed to recover for 30 min at 30°C, followed by plating onto LB agar supplemented with 25 µg/mL tetracycline, and incubation at 30°C overnight, or until clear colonies became visible. The absence of the gentamicin cassette was checked by patching single colonies simultaneously onto two LB agar plates, one containing 100 µg/mL gentamicin, or 25 µg/mL tetracycline. Both plates were incubated at 30°C overnight or until colonies appeared. Successful transformants resulted in the formation of colonies that grew on LB with tetracycline but did not grow in the presence of gentamycin.

The FLP recombinase expression plasmid pFLP3 was cured from the transformant cells using sucrose counter selection. Successful FLP recombinase transformants were streaked to single colony on YT-25% sucrose agar (10 g/L yeast extract, 20 g/L tryptone, 250 g/L sucrose, 18 g/L agar) for counter-selection against the FLP plasmid, incubating at 30°C for ~30–48 h. Surviving colonies were then re-streaked onto fresh YT-25% sucrose plates and incubated at 30°C for 16 h to remove any enduring *sacB*-containing cells. Single colonies were simultaneously patched onto two different LB agar plates, one containing 25% sucrose, and one containing tetracycline. Successfully cured cells resulted in colonies that were able to grow in the presence of sucrose, but not tetracycline. The final colonies were further verified for successful removal of the gentamycin resistance cassette by whole-genome resequencing.

### Salicyl alcohol degradation pathway design, synthesis, and integration

The SA degradation operon was amplified from *Pseudomonas* sp. GM16 genomic DNA using primers SA-Reg FWD and SA-Reg REV (Table 4) and polymerase Q5 (New England Biolabs, Massachusetts, USA). The destination plasmid pGW44 (34) was linearized using primers pGW44 FWD and pGW44 REV. The SA pathway was then introduced into pGW44 using the Gibson Assembly Cloning Kit (New England BioLabs), creating plasmid pJM455. The complete pathway was then genomically integrated into target strains using the BxB1 *attB* site, followed by the removal of the kanamycin selection marker, as described previously (34).

**TABLE 4:** Primers used in this study

Primer name	Primer sequence	Use
SA-Reg FWD	5′-TCATCCAAGTCTTCAATTGCAATCCTCAGAATGGATAAGGCTGGTC-3′	Cloning of pJM455
SA-Reg REV	5′-GTCCTCGAGTCTAGACCAGCTGATGTCAATCGCTGAAGAGATCAAAC-3′	Cloning of pJM455
pGW44 FWD	5′-CATCAGCTGGTCTAGACTCGAG-3′	Cloning of pJM455
pGW44 REV	5′-GGATTGCAATTGAAGACTTGGATG-3′	Cloning of pJM455
SC_BC_fwd_v2	5′-GTCTCGTGGGCTCGGAGATGTGTATAAGAGACAGGATGTCCACGAGGTCTCT-3′	Barcode amplification
SC_BC_rev_v2	5′-TCGTCGGCAGCGTCAGATGTGTATAAGAGACAGGTCGACCTGCAGCGTACG-3′	Barcode amplification

### Growth analysis

To measure growth kinetics with specific carbon sources, strains were grown to saturation in an M9 minimal medium with 1 g/L glucose as the sole carbon source. Saturated cultures were centrifuged for 3 min at 8,000 × *g*, washed with M9 minimal medium without carbon, and then diluted 1:100 into 100 µL fresh medium containing the indicated carbon source. Cultures were grown at 30°C in 96-well plates in a BioTek Epoch 2 shaking incubator (Agilent, Santa Clara, CA) for 72 h.

### Wild-type and mutant barcoding

To track and differentiate strains after eventual inoculation and growth on plant roots, a set of random DNA barcodes was also integrated into each genome. A plasmid library containing an R4 *attB* site and a stretch of 20 random nucleotides was synthesized (Biomatik, Ontario, Canada). The resulting barcode library was integrated into the R4 *attB* site of otherwise wild-type strains, containing only the landing pad, or strains with the SA catabolic pathway already integrated into the BxB1 *attP* site. The full libraries, which contained several million barcodes each, were subsampled to achieve an estimated library size of 10,000 barcodes per strain by spreading a dilution series on large LB agar plates and subsequently harvesting 10,000 colonies by washing a corresponding number of plates.

### Proteomics analysis

#### Cultivation

For proteomic analysis, wild-type and mutant strains were grown in test tubes containing 10 mL M9 medium supplemented with 0.1% glucose, 0.1% salicyl alcohol, or a combination of the two. A 5% salicyl alcohol stock solution was prepared in absolute ethanol and was added to the empty test tubes at the outset of media preparation, to allow evaporation of the ethanol before the addition of the M9. Glucose overnight cultures of the strains were washed twice in M9 without an energy source, inoculated into the test tubes, and finally incubated under shaking (30°C, 250 RPM) until reaching OD 0.5. Then, cells were harvested by centrifugation at 8,000 × *g* for 5 min, the supernatant was aspirated, and the cell pellet was immediately frozen at −80°C until further processing.

#### Cell lysis and protein extraction and digestion

Cell pellets were solubilized with 500 µL of lysis buffer (4% sodium dodecyl sulfate wt/vol in 100 mM Tris-HCl, pH 8.0). Samples were vortexed and then disrupted by bead beating for 5 min with 0.15 mm zirconium oxide beads at a 3:1 vol ratio of sample to beads. Samples were then placed in a heat-block for 10 min at 90°C. Approximately 400 µL of cell lysates were transferred to fresh Eppendorf tubes after centrifugation for 3 min at 21,000 × *g*. Protein concentration was measured using a NanoDrop One^C^ instrument (Thermo Scientific). Each sample was adjusted to 10 mM dithiothreitol (DTT) and incubated at 90°C for 10 min. Following DTT addition, samples were then adjusted to 30 mM iodoacetamide to prevent the reformation of disulfide bonds and incubated in the dark for 15 min. To isolate proteins, the protein aggregation capture method was employed (41). Briefly, Ser-Mag beads and crude lysates were added to fresh Eppendorf tubes at a 1:1 protein-to-beads ratio, precipitated, and captured by adjusting to 70% (vol/vol) liquid chromatography-mass spectrometry (LC-MS) grade ACN, then washed with 1 mL ACN and 1 mL of 70% LC-MS grade ethanol. Sequence-grade trypsin solution was added to a 1:75 (wt/wt) ratio of protein to trypsin and then additional Tris buffer was added to a final additional volume of 200 µL. Trypsin digestion was performed overnight at 37°C under constant shaking at 600 rpm using an Eppendorf Thermomixer (Thermo Scientific). Proteins were digested a second time using the same protein-to-trypsin ratio as before, but this time incubated for 3 h at 37°C under constant shaking at 600 rpm. After protein digestion, each sample was then adjusted to 0.5% formic acid (vol/vol) followed by vortexing and incubation at room temperature for 10 min. Each sample was then centrifuged at 21,000 *× g* for 10 min and supernatants were transferred on top of pre-equilibrated 10 kDa MW cutoff Vivaspin 500 filters. Tryptic peptide flowthroughs were then collected after centrifugation at 12,000 × *g* for 10 min. Peptide concentrations were measured using the same Nanodrop instrument as before and transferred to autosampler vials for liquid chromatography-tandem mass spectrometry (LC-MS/MS) measurement.

#### LC-MS/MS

Peptide mixtures were analyzed using two-dimensional liquid chromatography (LC) on an Ultimate 3000 RSLCnano system (Thermo Fisher Scientific) coupled with a Q Exactive Plus mass spectrometer (Thermo Fisher Scientific). For each sample, aliquots equivalent to 2 µg of peptides were injected into an in-house built strong cation exchange (SCX) Luna trap column (5 µm, 150 µm × 50 mm; Phenomenex, USA) followed by a nanoEase symmetry reversed-phase (RP) C18 trap column (5 µm, 300 µm × 50 mm; Waters, USA) and washed with an aqueous solvent. Cellular peptide mixtures were separated and analyzed across one SCX fraction by eluting the peptides from the SCX column with a volume plug of 500 mM ammonium acetate followed by a 90 min organic gradient (250 nL/min flow rate) to separate peptides across an in-house pulled nanospray emitter analytical column (75 µm × 350 mm) packed with 35 cm of C18 Kinetex RP C18 resin (1.7 µm; Phenomenex, USA). Mass spectra were acquired with the Q Exactive Plus instrument in a top 10 data-dependent acquisition setup. MS spectra were collected within 300 to 1,500 m/z with an automatic gain control (AGC) target value of 3 × 10^6^ at a resolution of 70,000 with a maximum injection time (IT) of 25 ms. Precursor ions with charge states ≥2 and ≤5 and intensity threshold of 1.6 × 105 were isolated using a 1.6 m/z isolation width for higher-energy C-trap collision dissociation with a normalized collision energy of 27 eV. MS/MS spectra were acquired at a resolution of 17,500 at m/z 200 with an AGC target value of 1 × 10^5^ and a maximum IT of 50 ms. Dynamic exclusion was set to 20 s to avoid repeated sequencing of peptides.

Each MS raw data file was processed by the SEQUEST HT database search algorithm and confidence in peptide-to-spectrum (PSM) matching was evaluated by Percolator (42) using the Proteome Discoverer v2.2 software. Peptides and PSMs were considered identified at q < 0.01 and proteins were required to have at least one unique peptide sequence. Protein relative abundance values were calculated by summing together peptide-extracted ion chromatograms. Protein abundances were normalized by LOESS and median central tendency procedures were performed on log_2_-transformed values by InfernoRDN (43). Differential protein abundances were identified through pairwise comparisons, with statistical significance assessed using a Student’s *t*-test. To control for multiple testing and reduce the likelihood of false positives, a permutation-based false discovery rate method was applied to calculate q-values using the Perseus computational platform (44).

#### Ortholog analysis for cross-species comparison

Ortholog groups were constructed with OrthoMCL using pre-configured workflows at the VEuPathDB Galaxy site. In brief, all-versus-all BLASTP and the OrthoMCL algorithm were used to assign each organism-encoded protein to OrthoMCL groups (version OG6r1) with a 1e-05 expectation value cutoff for BLASTP and a four main inflation value for the clustering algorithm MCL.

### Metabolite analysis

Salicin and salicyl alcohol of the PtrXB38-OE and control plants were extracted from ~150 mg of frozen powdered root tissue twice overnight with 2.5 mL of 80% ethanol. Sorbitol (75 µL of 1 mg/mL aqueous solution) was added to the first extract as an internal standard (38). The two extracts were combined, and a 1 mL aliquot was dried under nitrogen for analysis. The dried extracts were silylated to produce trimethysilyl derivatives by dissolving in 500 µL of silylation grade acetonitrile (Thermo Scientific, TS20062), followed by addition of 500 µL of N-methyl-N-trimethylsilyltrifluoroacetamide with 1% trimethylchlorosilane (Thermo Scientific, TS48915) and heated for 1 h at 70°C. After 2 days, 1 µL was injected into an Agilent Technologies 7890A GC coupled to a 5975C inert XL MS configured as previously described but with the following modification. Gas (He) flow was 1.20 mL per minute. Metabolite peaks were extracted using key mass-to-charge (m/z) selected ions to minimize interference with co-eluting metabolites and quantified as previously described, scaling back to the total ion chromatogram and normalizing to internal standard recovered, volume analyzed, and mass extracted.

### Differential localization experiments

#### Plant inoculation and incubation

Combinations of barcoded strains were created by pelleting, washing, and resuspending glucose-grown overnight cultures (30°C) in sterile, distilled water to OD 1, and then mixing them in equal amounts. *P. trichocarpa* BESC819, *P. tremula* x *P. alba* “INRA 717-1B4,” and the *PtrXB38-OE P. tremula* x *P. alba* “INRA 717-1B4” were propagated according to previously published procedures (45). In brief, sterile shoot tips were grown in tissue culture until root establishment (25°C, 16 h photoperiod). Then, plants similar in size and development were chosen, 5 mL of microbe combination was mixed into 150 cm^3^ calcined clay for each plant, and the root systems were placed in the clay and gently buried. After an incubation time of 21–28 days in a closed system magenta box (same conditions as specified above), root systems were lifted from the clay, loosely attached clay particles removed, and the roots frozen at −20°C until further processing. For differential localization analysis, nine sampling locations were devised, consisting of a cross of three structural categories; primary, secondary, and tertiary, with three root regions; root tips, midzone, and root hairs (Fig. 5A) (46).

#### Nucleic acid extraction

According to their development, roots were dissected into primary (oldest and thickest roots), secondary (first branching roots, medium size, and age), and tertiary (youngest and thinnest). Furthermore, three sample types were collected for each structural category, i.e., tips (root tips of the structural category), hair roots (originating from the surface of the corresponding structure), and segments (root mass from the middle of the structure, whereby hair roots were removed from the surface). For each combination, 0.2 g of root mass was pooled for nucleic acid extraction and an initial pulverization step was conducted. This step consisted of freezing the tubes containing the root fragments in liquid nitrogen and bead-beating them three times for 1 min at 30 Hz using a TissueLyser II (Qiagen) and the steel beads included in the DNeasy Plant Pro Kit (Qiagen) with intermittent refreezing in liquid N_2_. Then, the pulverized root material was used as the regular input for said kit according to the manufacturer’s instructions. Bacterial mixtures used for inoculating plant experiments were extracted using the DNeasy Blood and Tissue Kit (Qiagen) according to the manufacturer’s recommendations.

### Library preparation, sequencing, and data analysis

Microbial genomic DNA obtained from plant dissects and inoculum mixtures were amplified to enrich the barcode locus using primers suitable for sequencing adapter attachment depending on the intended sequencer (Table 4). Amplicons were then pooled at equimolar concentration and sequenced using an in-house Illumina MiSeq sequencer, as well as commercially via Illumina NovaSeq technology (VANTAGE, Vanderbilt University, Nashville, TN). From the resulting data, barcodes were extracted from sequencing reads by Bartender v1.1 (47), summarized, and then analyzed in R (v 4.0.3) using the tidyverse package (v 1.3.0) and a custom visualization script.

## Data Availability

Raw sequencing reads generated and analyzed for this study can be downloaded from the NCBI Sequence Read Archive under Bioproject PRJNA1054559. Custom analysis scripts are available at https://github.com/s-christel/salicylate_barcode_experiment. All proteomics spectral data in this study were deposited at the ProteomeXchange Consortium via the MassIVErepository (https://massive.ucsd.edu/). The ProteomeXchange project identifier is PXD048223 and the MassIVE identifier is MSV000093756.

## SUPPLEMENTAL MATERIAL

Suppemental figures mbio.03016-24-s0001.pdf Figures S1 to S6.

ASM does not own the copyrights to Supplemental Material that may be linked to, or accessed through, an article. The authors have granted ASM a non-exclusive, world-wide license to publish the Supplemental Material files. Please contact the corresponding author directly for reuse.
