# Supplementary material for: Catabolic pathway acquisition by rhizosphere bacteria readily enables growth with a root exudate component but does not affect root colonization
Source: mBio. 2024 Dec 11;16(1):e03016-24. doi: 10.1128/mbio.03016-24 (PMC11708038; doi:10.1128/mbio.03016-24)
Supplement: Suppemental figures — Figures S1 to S6. [file mbio.03016-24-s0001.pdf]

Supplementary Information

Catabolic pathway acquisition by rhizosphere bacteria readily enables growth with a component of the root exudate but does not affect root colonization

Stephan Christel[1], Alyssa A. Carrell[1], Leah H. Hochanadel[1], Manuel I. Villalobos Solis[1], Paul E. Abraham[1], Sara S. Jawdy[1], Julie E. Chaves[1], Nancy L. Engle[1], Timkhite-Kulu Berhane[1], Tao Yao[1], Jin-Gui Chen[1], Wellington Muchero[1*], Timothy J. Tschaplinski[1], Melissa A. Cregger[1], and Joshua K. Michener[1,†]

[1]Biosciences Division, Oak Ridge National Laboratory, 1 Bethel Valley RD, Oak Ridge, TN 37831, USA

[†] To whom correspondence should be addressed: michenerjk@ornl.gov
* Deceased

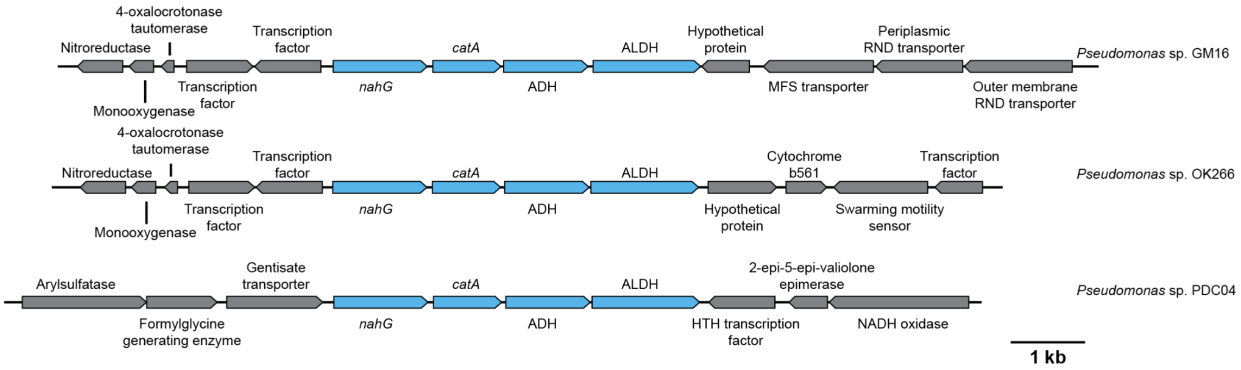

Figure S1: Three distantly-related SA-catabolizing strains contain homologous SA catabolic operons. Homologous *nahG* sequences were identified through BLAST searches and the flanking genome regions compared. A *nahG* homolog was also found in *Pseudomonas* sp. GM41 but not in an equivalent operon. No homologs were found in the other genome-sequenced strains. No genome is available for *Pseudomonas* sp. OK64, so this strain was not used in the analysis. For comparison, the four NahG enzymes have approximately 80% pairwise amino acid identity.

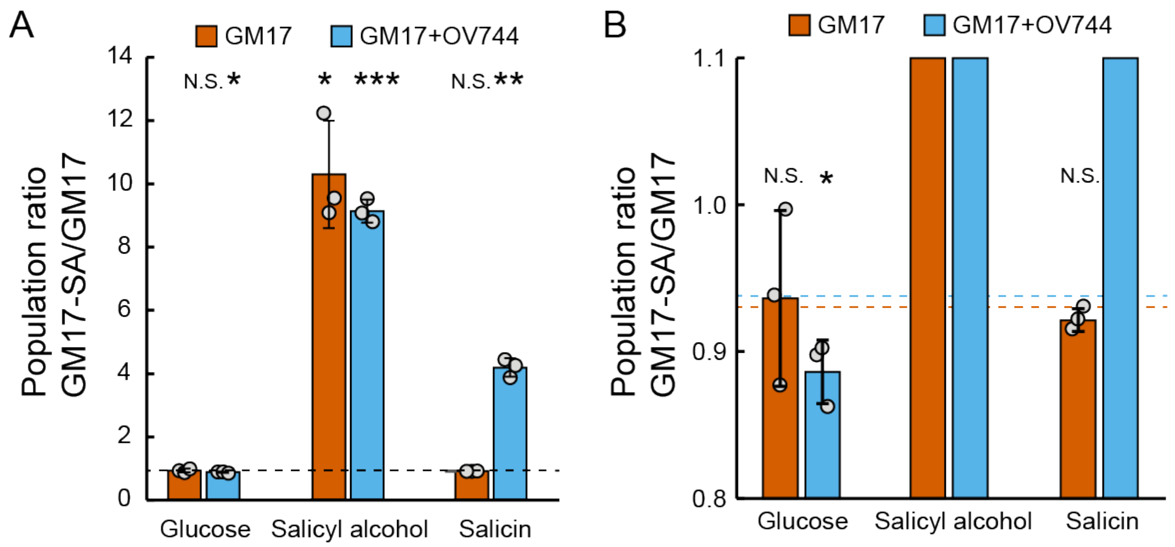

Figure S2: The SA catabolic pathway provides an advantage during growth in liquid culture with SA. A mixture of barcoded wild-type and engineered GM17 strains were grown in MOPS minimal medium with the indicated carbon source in the presence or absence of OV744. Population ratios before and after growth were calculated by barcode amplicon sequencing. The dashed line shows the population ratio for the inoculum. Data in panel B is the same as in A, but expanded around a population ratio of 1.0. Error bars show one standard deviation, calculated from three biological replicates. *: $p<0.05$; **: $p<0.01$; ***: $p<0.001$; N.S.: not significant ($p>0.05$).

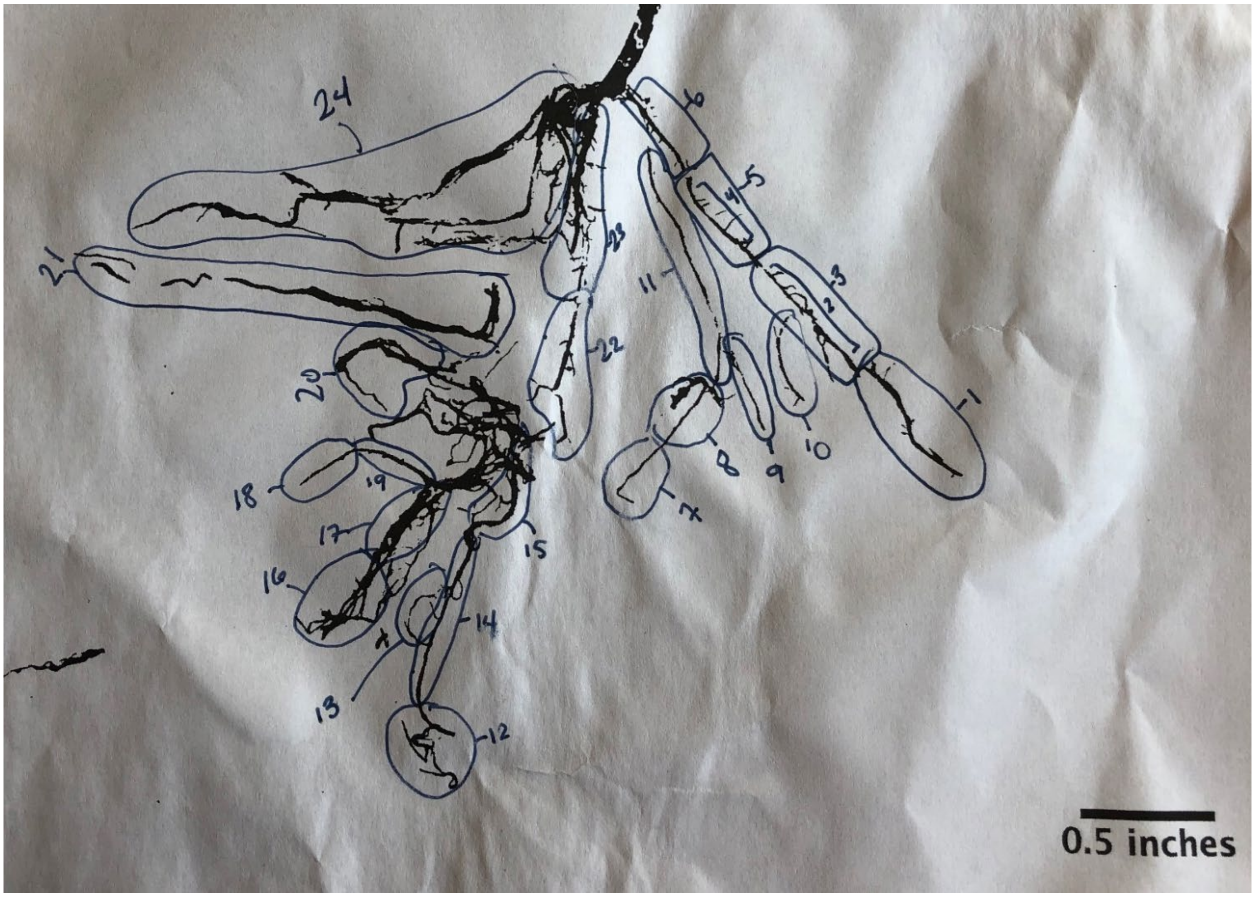

Figure S3: Root dissection optimization tested a range of root sizes and orders. A single tissue cultured *Populus trichocarpa* root system previously inoculated with barcoded wild-type *Pseudomonas* sp. GM17 was isolated, imaged, and dissected as indicated. Genomic DNA was extracted from each root segment and barcodes were amplified by PCR, as shown in Figure S3.

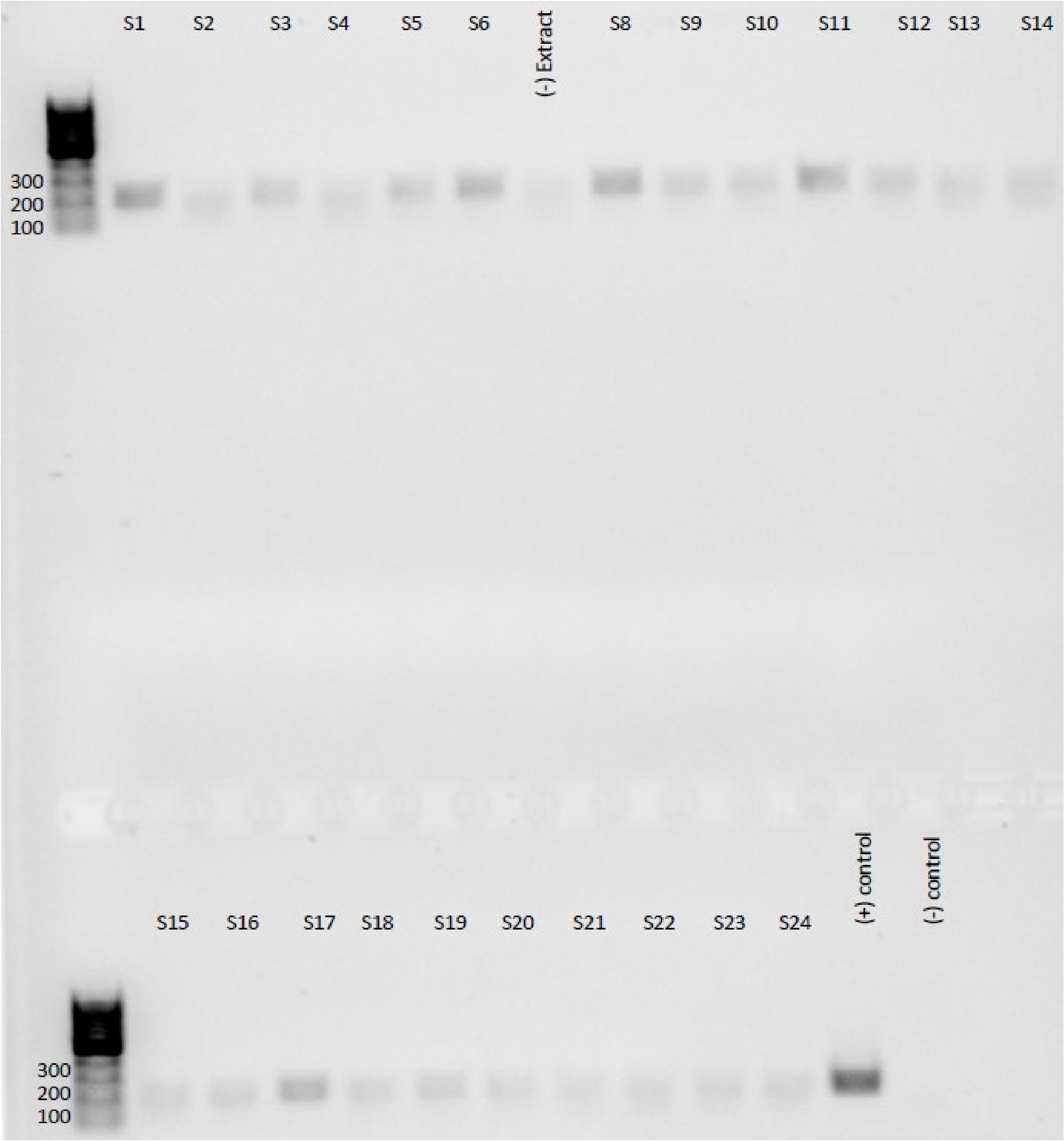

Figure S4: Barcode amplification from root segments. Sample IDs are as indicated in Figure S3. Barcodes were amplified by PCR and sequenced.

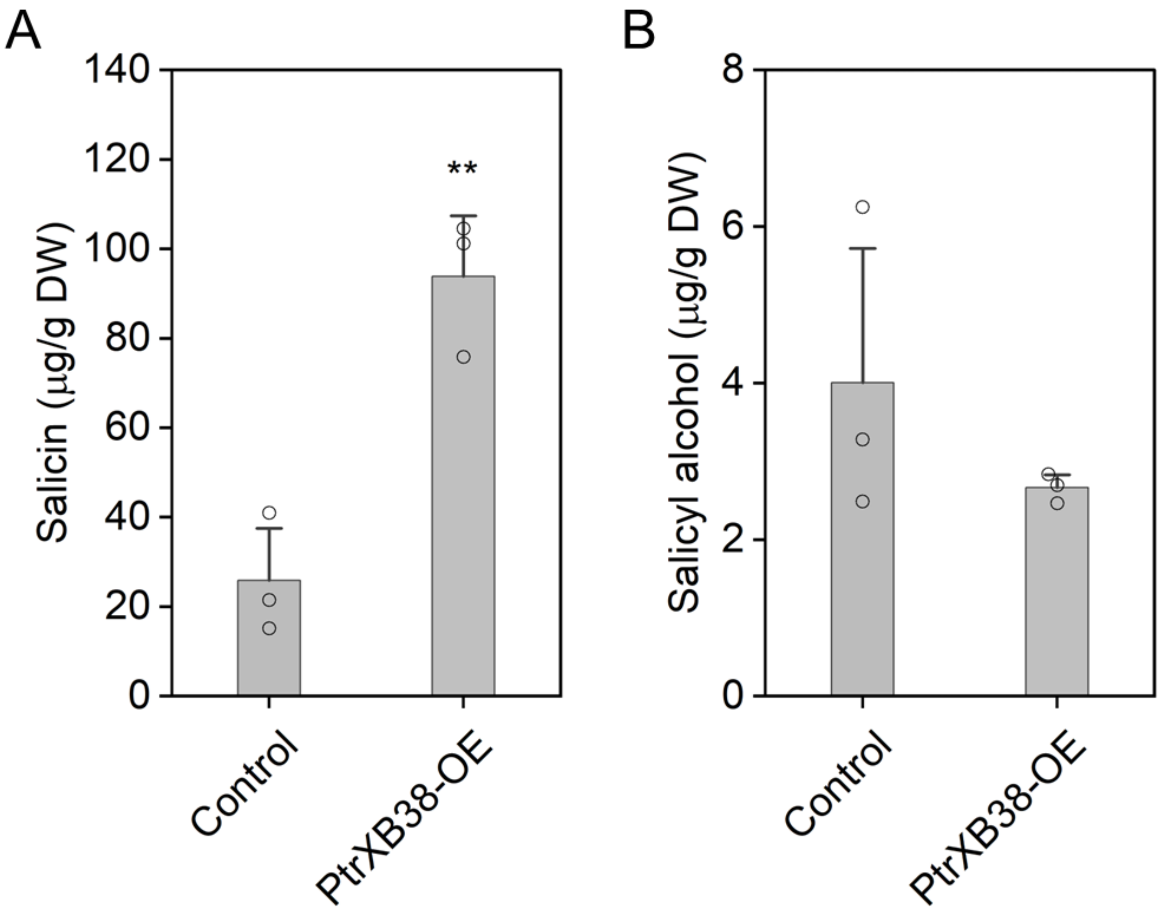

Figure S5: Overexpression of PtrXB38 increases production of salicin in the roots of poplar (*Populus tremula* x *Populus alba*). Roots from 2-month-old empty vector control and PtrXB38-OE transgenic plants were analyzed by GC-MS. Metabolite concentrations are relative to sorbitol, the internal standard. Bar charts represent mean ± SE (n= 3 independent plants), and double asterisk (**) represents significant difference between groups ($P<0.01$) by the Student's *t*-test.

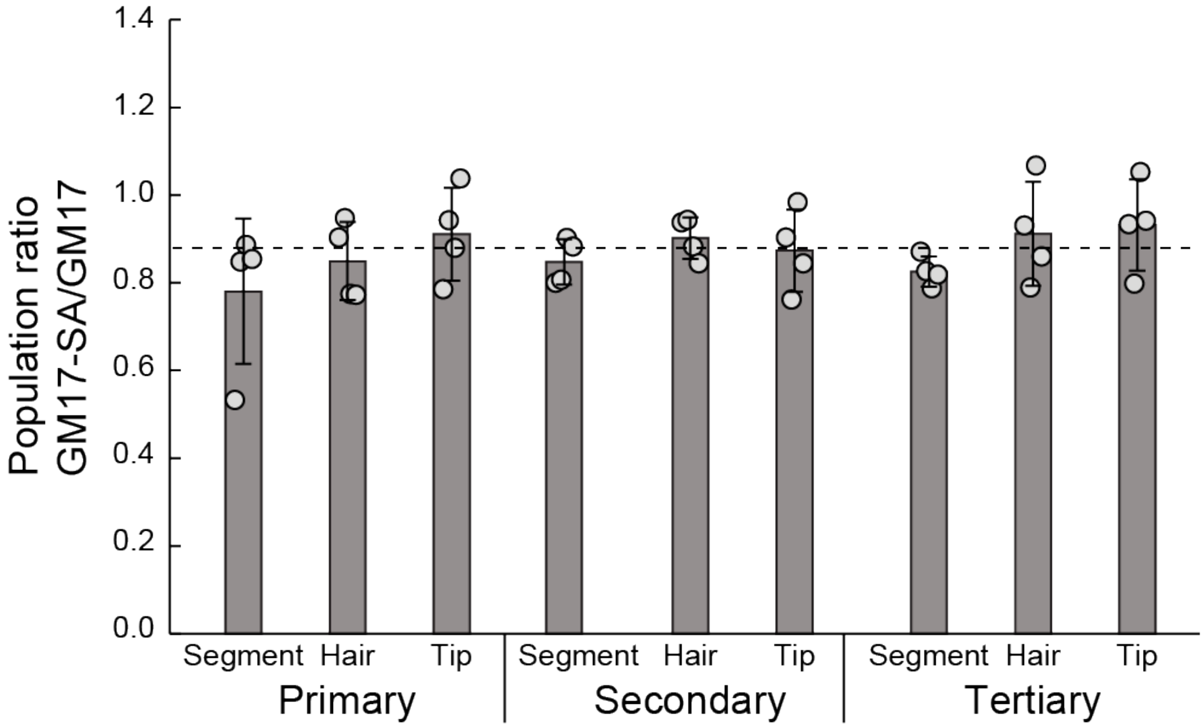

Figure S6: SA catabolism does not provide a fitness advantage to GM17-SA during growth on XBAT35 trees. Experiments were conducted as described in Figure 5D except without the addition of *Rahnella* sp. OV744.The dashed line shows the population ratio of the inoculum. Error bars show one standard deviation, calculated from the four biological replicates shown. None of the population ratios differed significantly from the initial inoculum ($p<0.05$).
